# Partial Anomalous Pulmonary Venous Connection in Turner Syndrome: Small Patients, Big Implications

**DOI:** 10.1016/j.cjcpc.2025.07.002

**Published:** 2025-07-31

**Authors:** Ashish H. Shah

**Affiliations:** Section of Cardiology, Department of Internal Medicine, Rady Faculty of Health Sciences, University of Manitoba, Winnipeg, Manitoba, Canada

**Keywords:** anomalous pulmonary vein, Turner syndrome, impact

I read with great interest the article by Tenisch *et al.*^1^ on partial anomalous pulmonary venous connection (PAPVC) in Turner syndrome (TS) (79 patients; mean age 25 years [5-67 years]). TS is known to have a high prevalence of PAPVC, with cardiac magnetic resonance imaging studies reporting rates of 15%-25%.^2^ However, PAPVC can also occur in the general population, either in isolation or in association with other congenital anomalies. In our previous study on isolated PAPVC, TS was present in only approximately 11% of cases—similar to associations with bicuspid aortic valve and coarctation of the aorta.^3^ Tenisch *et al.* rightly emphasize the limitations of transthoracic echocardiography, which often fails to detect PAPVC. Given this, patients with TS should undergo cross-sectional imaging to evaluate for PAPVC and other anomalies such as coarctation.

Physiologically, PAPVC produces a left-to-right shunt akin to an atrial septal defect (ASD), leading to right heart volume loading. The magnitude of shunting depends on the diameter and compliance of the anomalous pulmonary vein, the right heart’s compliance, and the pressure gradient across the anomalous pulmonary vein connecting the pulmonary artery to the right atrium. Although the patients in the current series were young, aging is typically associated with reduced left ventricular compliance—particularly in the presence of bicuspid aortic valve–related dysfunction, while right ventricular (RV) compliance is relatively preserved. This imbalance may lead to increased left-to-right shunting, especially during physical exertion or athletic activity. Unlike ASD, PAPVC lacks an interatrial communication that might decompress the RV under pressure. Consequently, if pulmonary hypertension develops, patients with PAPVC and an intact septum may experience worse outcomes compared with those with ASD.

Only 1 patient in the series had significant RV dilation requiring surgical repair, but the cohort was relatively young, and no long-term data were presented. In our cohort of 53 patients with isolated PAPVC (mean age 47 ± 19 years), approximately 15% developed RV dilation (right ventricular end-diastolic volume, indexed >150 mL/m^2^) and several progressed to pulmonary hypertension.^3^ This highlights the need for lifelong surveillance. In TS, short stature and low body surface area further necessitate careful indexing of RV volumes, as modest absolute dimensions may mask significant dilation.

Finally, ratio between pulmonary (Qp) and systemic flow (Qs)—commonly used to quantify shunt—can be misleading, as a normal ratio may still reflect high absolute pulmonary flow if systemic output is elevated. Hence, quantifying the absolute pulmonary flow volume offers a more meaningful clinical measure.

In summary, patients with TS should undergo routine cross-sectional imaging to detect PAPVC, even when transthoracic echocardiography is unremarkable. Lifelong monitoring of RV volume and pressure, with proper indexing, is essential.
